# The novel IDO-1 inhibitor 3-047 combined with icaritin ameliorates neuroinflammation and diabetes-associated cognitive dysfunction with suppression of TLR4/MyD88/NF-κB signaling

**DOI:** 10.3389/fimmu.2026.1704307

**Published:** 2026-01-26

**Authors:** Tingmei Mo, Xia Zhuang, Wenxue Zhao, Baohua Wang, Yuting Wang, Wenjie Ji, Dongguang Liu, Guimin Zhang, Ru Yao, Yan Xu, Jingchun Yao

**Affiliations:** 1 School of Pharmacy, Qingdao University, Qingdao, Shandong, China; 2 School of Pharmacy, Shandong University of Traditional Chinese Medicine, Jinan, Shandong, China; 3 Department of Pharmacy, Affiliated Hospital of Changchun University of Chinese Medicine, Changchun, Jilin, China; 4 School of Medicine and Pharmacy, Ocean University of China, Qingdao, Shandong, China; 5 School of Traditional Chinese Medicine, Guangdong Pharmaceutical University, Guangzhou, Guangdong, China; 6 State Key Laboratory of Integration and Innovation of Classic Formula and Modern Chinese Medicine, Lunan Pharmaceutical Group Co., Ltd, Linyi, Shandong, China; 7 Lunan Better Pharmaceutical Co., Ltd, Linyi, Shandong, China

**Keywords:** cognitive dysfunction, diabetes, icaritin, IDO-1 inhibitor, neuroinflammation

## Abstract

**Background:**

Diabetes-associated cognitive dysfunction (DACD) is one of the common chronic complications of diabetes mellitus in the central nervous system. Given that current therapeutic agents are limited, exploring novel therapeutic agents is particularly important. The novel IDO-1 inhibitor 3–047 and icaritin (ICT) demonstrate efficacy in ameliorating neuroinflammation and cognitive dysfunction, and their combination exhibits certain hypoglycemic effects. However, the impact of this combination therapy on DACD remains unclear.

**Methods:**

The db/m mice were used as the control group, and the db/db mice were divided into the model group, the combined low-dose group, the combined medium-dose group, the combined high-dose group, and the metformin group. Drug treatment was administered for 16 weeks. Blood glucose, body weight and homeostatic model assessment of insulin resistance were detected as the basic indexes. The Morris water maze was used to evaluate the spatial learning and memory ability of mice. Neuronal damage, apoptosis and degeneration were observed by H&E staining, Nissl staining, TUNEL staining and Fluoro-Jade C staining. Additionally, ultrastructural changes of the hippocampus were observed by transmission electron microscopy. Serum inflammatory factors expression was detected via ELISA, while microglial expression was assessed by immunofluorescence. Protein expression related to the TLR4/MyD88/NF-κB pathway, neuroinflammation, cognitive impairment, synaptic damage, and neuronal apoptosis was analyzed through Western blotting.

**Results:**

Combined therapy with 3–047 and ICT not only improved spatial learning and memory deficits in db/db mice but also modulated the expression of proteins associated with cognitive dysfunction. It inhibited neuronal apoptosis and degeneration, alleviated hippocampal ultrastructural damage, and simultaneously reduced the expression of TLR4/MyD88/NF-κB-related proteins and the occurrence of neuroinflammation.

**Conclusion:**

3–047 combined with ICT reduces neuronal apoptosis and neuroinflammation and improves cognitive dysfunction in diabetic mice, and these effects are potentially associated with downregulation of the TLR4/MyD88/NF-κB pathway.

## 1 Introduction

Diabetes mellitus is a metabolic disease characterized by persistent hyperglycemia, and its progression is often accompanied by a variety of complications. Among them, diabetes-associated cognitive dysfunction (DACD) is one of the more common central nervous system complications, which mainly manifests as a decline in learning ability, attention and spatial memory. Studies have shown that patients with diabetes have an approximately 1.25- to 1.91-fold increased risk of developing cognitive impairment compared with the general population (1). The disease is broadly categorized into three stages depending on the severity of the condition: diabetes-associated cognitive decline, mild cognitive impairment, and dementia (2). At the stage of dementia, patients almost lose their ability to perform activities independently. Currently, drugs used to treat DACD are limited and have some side effects, so it is important to find effective treatment strategies.

It is noteworthy that DACD shares certain pathological similarities with Alzheimer’s disease, both involving insulin resistance, amyloid beta (Aβ) deposition, and hyperphosphorylation of the microtubule-associated protein tau (3, 4). Insulin resistance impairs brain cell signaling, weakens brain plasticity, and disrupts hippocampal synaptic ultrastructure (5, 6), leading to diminished learning and memory functions. Additionally, abnormal processing of the amyloid precursor protein (APP) generates oligomeric Aβ, which aggregates into amyloid plaques. Plaque accumulation not only causes synaptic degeneration and loss in neighboring neurons but also disrupts the dynamic equilibrium between toxic Aβ oligomers and inert fibrils. This promotes the spread of harmful substances, inducing neurotoxicity, synaptic dysfunction, and inflammatory responses (7–9). Furthermore, the tau protein, which maintains microtubule stability, undergoes excessive phosphorylation under pathological conditions. This leads to the formation of neurotoxic aggregates (10) that accelerate neuronal loss (11), ultimately resulting in cognitive impairment.

Neuroinflammation plays a key role in the development and progression of cognitive dysfunction in diabetes. Among them, microglia and astrocytes are the core cells that regulate neuroinflammation (12). Persistent hyperglycemia not only induces the conversion of microglia to a pro-inflammatory phenotype (M1), which is manifested by enhanced phagocytosis and increased release of pro-inflammatory factors (13, 14). The hyperglycemic state also activates Toll-like receptor 4 (TLR4) on the surface of microglial cells (15), which leads to the sustained amplification of the inflammatory response. Activation of TLR4 further recruits myeloid differentiation factor 88 (MyD88), subsequently triggering the downstream cascade of the nuclear factor κB (NF-κB) response. This promotes the expression of inflammatory cytokines such as tumor necrosis factor (TNF-α), interleukin-1β (IL-1β), interleukin-6 (IL-6), and chemokines, ultimately leading to neuronal injury and synaptic dysfunction (15–17). This suggests that modulation of the TLR4/MyD88/NF-κB pathway and neuroinflammation may be a potential target for the treatment of DACD.

Indoleamine 2,3-dioxygenase 1 (IDO-1) is the rate-limiting enzyme of tryptophan-kynurenine metabolism. Activation of IDO-1 promotes the production of a variety of tryptophan catabolic metabolites with important neurotoxic properties, such as quinolinic acid and 3-hydroxykynurenine, within the central nervous system and microglia, which may further induce neuroinflammation (18). Studies have shown that inhibition of IDO-1 expression can effectively alleviate diabetic neuroinflammation induced by a high-fat diet (19) and improve cognitive dysfunction (20). Therefore, inhibition of IDO-1 expression is considered as a potential target for diabetic cognitive dysfunction. Compound 3–047 is a novel IDO-1 inhibitor. Although detailed pharmacokinetic data for 3–047 remain proprietary, preliminary laboratory studies have demonstrated its safety and efficacy *in vivo*. In summary, 3–047 holds promise as a potential therapeutic agent for diabetic cognitive impairment. Icaritin (ICT) is mainly derived from the hydrolysis of icariin, a flavonoid compound in the traditional Chinese medicine *Epimedium* (21). ICT not only improves blood glucose and insulin resistance in diabetic mice (22), but also exerts neuroprotective effects through anti-inflammatory and antioxidant mechanisms in models of central nervous system-related diseases (23).

Meanwhile, studies by Li et al. indicate that the combination therapy of 3–047 and ICT still exhibits certain hypoglycemic effects (24). However, its therapeutic efficacy in diabetic-associated cognitive dysfunction (DACD) has not been established. Therefore, we evaluated the combined treatment of 3–047 and ICT to determine its potential benefits in DACD.

## 2 Materials and methods

### 2.1 Reagents

3-047 (220502) and Icaritin (220507) were purchased from Shandong New Era Pharmaceutical Co., Ltd. (Shandong, China), metformin hydrochloride enteric-coated tablets (20240118) were purchased from Guizhou Tian’an Pharmaceutical Co., Ltd. (Guizhou, China). TNF-α ELISA Kit (ml0020951), IL-1β ELISA Kit (ml301814), IL-6 ELISA Kit (ml098430), Mouse Insulin Assay Kit (ml001983) were purchased from Shanghai Enzyme-Linked Bio-Technology Co., Ltd. (Shanghai, China). Fluoro-Jade C Staining Kit (G3262) and Congo Red Staining Kit (G1535) were purchased from Beijing Solarbio Science & Technology Co., Ltd. (Beijing, China). Nissl staining solution (C0117), TUNEL staining kit (C1088), and anti-β-actin antibody were purchased from Beyotime Biotechnology (Shanghai, China). Antibodies Iba-1 (ab178846), iNOS (ab178945), Bax (ab32503), GAP43 (ab75810), GFAP (ab7260), PSD95 (ab238135), NeuN (ab177487), and p-Tau (ab32057) were purchased from abcam (Cambridge, UK). CD206 (24595), NF-κB (8242), p- NF-κB (3033), APP (2450), p-APP (6986), MyD88 (4283), TNF-α (3707), IL-1β (12242), IL-6 (12912) were purchased from Cell Signaling Technology (Danvers, MA, USA). TLR4 (sc-293072) and Bcl-2 (sc-7382) were purchased from Santa Cruz Biotechnology (Santa Cruz, CA, USA).

### 2.2 Animals and groups

Seven-week-old male db/m and db/db mice were purchased from Changzhou Cavens Laboratory Animal Co., Ltd. (License No: SCXK (Su) 2021-0013; Changzhou, China). All mice were housed under standard conditions (23 ± 3 °C, 12 h/12 h light-dark cycle) with ad libitum access to food and water. All animal-related procedures were approved by the Institutional Animal Care and Use Committee of the New Drug Pharmacology Center, Lunan Pharmaceutical Group Co., Ltd. (Approval No: HN-IACUC-2024-111).

**Figure 1:**
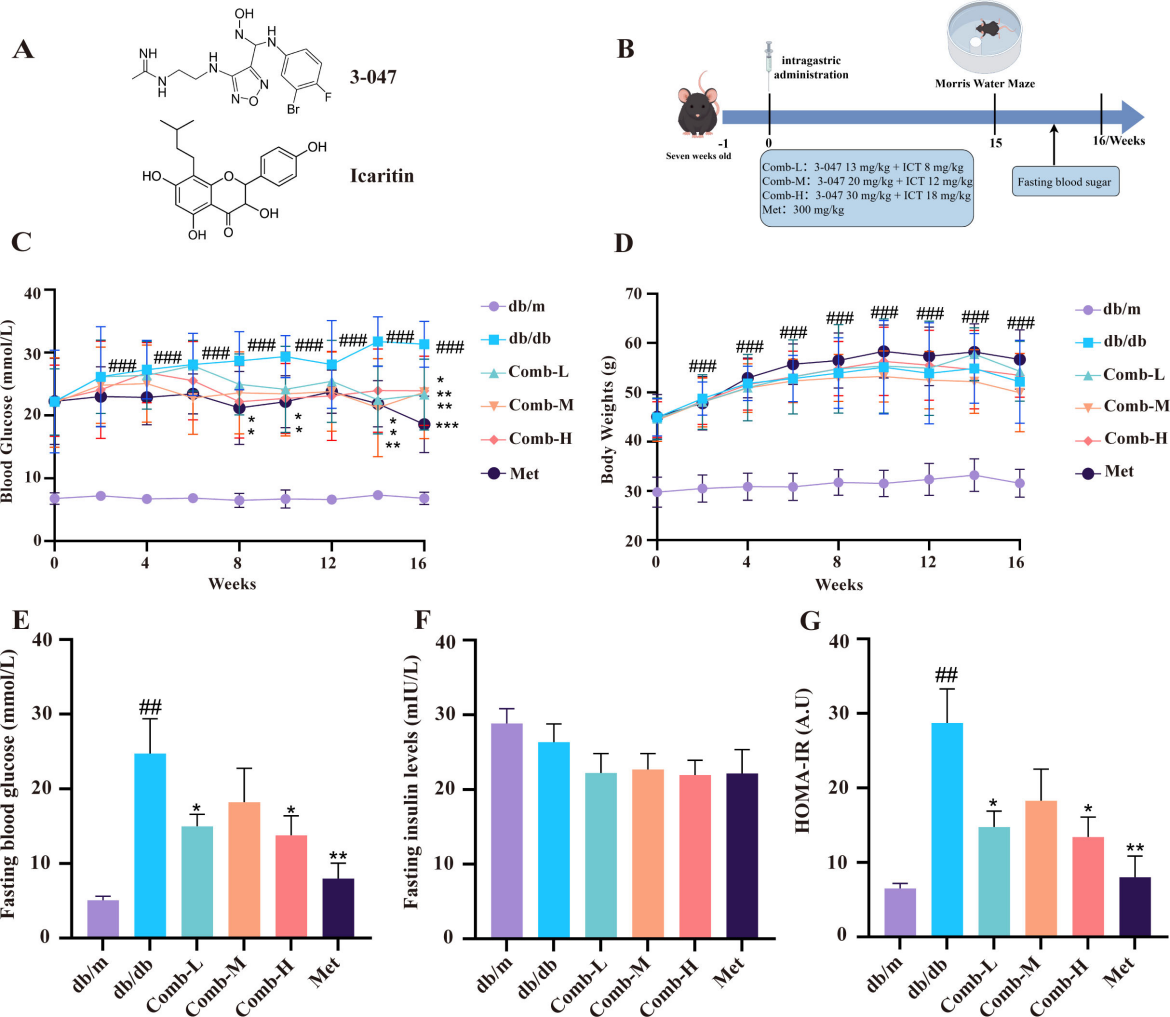
3–047 combined with ICT improves blood glucose, body weight, and insulin resistance in DACD mice. **(A)** Chemical structures of IDO-1 inhibitor 3–047 and Icaritin (ICT). **(B)** Experimental workflow diagram by Figdraw. **(C)** Blood glucose levels (n = 11 per group). **(D)** Body weight (n = 11 per group). **(E)** Fasting blood glucose levels (n = 6 per group). **(F)** Fasting insulin levels (n = 6 per group). **(G)** HOMA-IR (n = 6 per group). Data are presented as mean ± SD. Compared with db/m mice, ^###^*p* < 0.001, ^##^*p* < 0.01; compared with db/db mice, ^***^p < 0.001, ^**^p < 0.01, ^*^p < 0.05.

After one week of acclimatization, random blood glucose levels were measured via the tail vein. A glucose level > 16.7 mmol/L indicated the successful establishment of the diabetic model, at which point db/m mice weighed 24–34 g and db/db mice weighed 37–51 g. Subsequently, db/db mice were randomly assigned to five groups based on glucose levels and body weight using IBM SPSS Statistics for Windows Version 27.0 (IBM Corp., Armonk, NY, USA) to generate random numbers. The specific groupings (n = 11 per group) were as follows: the control group (db/m, purified water), the model group (db/db, purified water), the combined low-dose group (db/db, 3-047–13 mg/kg + ICT 8 mg/kg, Comb-L), the combined medium-dose group (db/db, 3-047–20 mg/kg + ICT 12 mg/kg, Comb-M), the combined high-dose group (db/db, 3-047–30 mg/kg + ICT 18 mg/kg, Comb-H) (24), and the metformin group (db/db, metformin 300 mg/kg, Met) (25, 26). Intragastric administration was performed for 16 consecutive weeks. Blood glucose and body weight were measured biweekly. The Morris water maze test was conducted at week 15. Following the final drug administration (**Figure 1B**), mice were fasted for 12 h and anesthetized via intraperitoneal injection of 5 mg/kg of Xylazine and Zoletil 50. After anesthetizing the mouse and collecting blood via the abdominal main vein, the thoracic cavity was incised and the right atrial appendage excised. Subsequently, a needle was inserted into the left ventricle, where 10 mL of physiological saline was first injected for perfusion. The brain was then removed and placed on an ice tray. Using a sterile scalpel blade, the brain was bisected. The cortex of the left hemisphere was dissected laterally to expose the hippocampus, and the hippocampus was gently lifted from the tail and dissected using forceps, then stored in a -80 °C freezer for subsequent marker detection. The right cerebral hemisphere was fixed either in 4% paraformaldehyde for subsequent histological analyses or in an electron microscopy fixative for ultrastructural examination.

### 2.3 Homeostatic model assessment of insulin resistance (HOMA-IR)

After 12 h of fasting, blood was collected from the tail vein to measure blood glucose levels in mice. Fasting insulin levels were determined using an ELISA kit, and values were obtained using a Multiskan GO 1510 microplate reader (Thermo Fisher Scientific, Vantaa, Finland). The insulin resistance index was calculated using the formula: HOMA-IR = fasting blood glucose level (mmol/L) × fasting insulin level (mIU/L)/22.5 (27).

### 2.4 Morris water maze (MWM)

Spatial learning and memory in diabetic mice were assessed using the Morris water maze (Jiangsu Saionsi Biotechnology Co., Ltd., Jiangsu, China) and evaluated with ANY-maze software (v7.3; Stoelting Co., Wood Dale, IL, USA). The MWM test was conducted according to the previous method with partial modifications (28). Mice were acclimated to the water maze room one day prior to testing to prevent stress. Purified water was added to the circular pool (120 cm in diameter) and maintained at a constant temperature of 23 °C. All experiments were conducted between 9:00 AM and 5:00 PM under dim lighting (< 100 lux). The entire experiment was divided into three phases: a 1-day platform visible phase, a 5-day platform hidden phase, and a 1-day spatial memory exploration period. The pool was divided into four quadrants, and an 8-cm diameter platform was positioned in the fourth quadrant. During the visible platform phase, the platform was raised 1 cm above the water surface. During the hidden platform phase, the platform was submerged 1.5 cm below the water surface. Each day, mice were placed in the water from four different quadrants. If a mouse failed to locate the platform within 60 s, it was manually guided to the platform and allowed to remain there for 15 s. For the spatial memory exploration period, the platform was removed, and mice entered the water from the second quadrant to locate the original platform position. During this period, swimming speed, total distance traveled, time spent in the target quadrant, and the number of platform crossings were recorded.

### 2.5 Hematoxylin and eosin (H&E) staining

Brain tissue fixed in 4% paraformaldehyde solution was removed, dehydrated, embedded, and sectioned into 4 μm slices. Sections were deparaffinized in xylene for 10 min, followed by rehydration through a graded ethanol series (100%, 95%, 85%, and 70% ethanol) for 5 min each, and immersion in distilled water for 2 min. Sections were subsequently stained with hematoxylin and eosin, dehydrated using a gradient of ethanol and xylene, and mounted. Finally, images were acquired and captured using the Leica DM2500 LED microscope and LAS X system (Leica Microsystems, Wetzlar, Germany) in a blinded manner.

### 2.6 Nissl staining

After dewaxing and rehydration, tissue sections were stained with Nissl stain at 37 °C for 1 h. Excess stain was rinsed off with distilled water, followed by nuclear counterstaining with hematoxylin. The sections were washed with tap water and rehydrated. After mounting with neutral resin, the images were acquired using the aforementioned Leica microscope system in a blinded manner and quantified using ImageJ software.

### 2.7 TUNEL staining

Paraffin sections were dewaxed and rehydrated. DNase-free proteinase K was added dropwise and incubated at 37 °C for 20 min. After washing with PBS, 50 μL of TUNEL staining solution was added and incubated at 37 °C in the dark for 1 hour. The sections were then washed three times with PBS, and counterstained with DAPI, sealed with an anti-fluorescence quencher. Images were acquired and captured with an IM-5FLD inverted microscope and CapStudio software (OPTIKA, Ponteranica, Italy) in a blinded manner, and quantified using ImageJ software.

### 2.8 Fluoro-Jade C (FJC) staining

After dewaxing and rehydration, the paraffin-embedded tissue sections were immersed in Reagent A from the FJC Kit for 5 min. Sections were then transferred to 70% ethanol for 2 min, followed by rinsing with distilled water for 2 min. Subsequently, sections were incubated in Reagent B for 10 min, washed with distilled water, and then incubated with Reagent C. Incubation was performed at room temperature in the dark for 10 min, followed by three washes with distilled water and counterstaining with DAPI. Finally, sections were mounted with an anti-fluorescence quencher. Images were captured in a blinded manner using the aforementioned IM-5FLD microscope system and quantified using ImageJ software.

### 2.9 Congo red staining

After dewaxing and rehydration, tissue sections were immersed in Highman Congo Red staining solution for 8 min, followed by 5 s of alkaline differentiation. Differentiation was immediately terminated by rinsing with tap water. Nuclei were then stained with Lillie-Mayer hematoxylin solution for 2 min, followed by washing in tap water for 10 min. Sections were subsequently dehydrated and cleared using a graded series of ethanol and xylene, and mounted with neutral resin. Finally, images were acquired in a blinded manner using the aforementioned Leica microscope system.

### 2.10 Immunofluorescence

Tissue sections were dewaxed and rehydrated, followed by antigen retrieval for 18 min. Sections were then incubated with 3% hydrogen peroxide for 10 min, followed by membrane permeabilization with 0.5% Triton X-100 in the dark for 10 min, and blocking at 37 °C for 1 h. Sections were incubated overnight at 4 °C with primary antibody Iba-1 (1:2000), followed by incubation at 37 °C for 2 h with the corresponding fluorescent secondary antibody. Subsequently, sections were counterstained with DAPI and mounted with an antifade mounting medium. Finally, fluorescence images were captured in a blinded manner using the aforementioned IM-5FLD microscope system and quantified using ImageJ software.

### 2.11 Transmission electron microscope (TEM)

Whole-brain perfusion was performed with 2% paraformaldehyde and 2.5% glutaraldehyde. Hippocampal tissue was excised and sectioned into approximately 1 mm³ fragments. Specimens were fixed at 4 °C in electron microscopy fixative. Subsequently, samples were post-fixed for 2 h at room temperature in the dark with 1% osmium tetroxide in 0.1 M PBS (pH 7.4). Tissues were rinsed three times with 0.1 M PBS, and sequentially dehydrated through a graded ethanol series (30%, 50%, 70%, 80%, 95%, and 100%), followed by two rinses in 100% acetone. The specimens were then embedded in epoxy resin overnight at 37 °C. The embedded blocks were polymerized at 60 °C for 48 h, sectioned into 70-nm ultrathin slices, and stained with uranyl acetate-saturated ethanol solution and lead citrate solution. Finally, the sections were observed using a TEM (HT7800, Hitachi, Japan) at 80 kV acceleration voltage.

### 2.12 ELISA

After anesthesia, blood was collected from mice and allowed to stand at room temperature for 2 h. The samples were then centrifuged at 4000 rpm and 4 °C for 10 min. The supernatant was collected, aliquoted, and used to detect serum levels of TNF-α, IL-1β, and IL-6 according to the ELISA kit instructions. Absorbance was measured using the Multiskan GO 1510 microplate reader (Thermo Fisher Scientific, Vantaa, Finland) to obtain the values.

### 2.13 Western blotting

Trim the hippocampal tissue into small pieces and homogenize using RIPA lysis buffer containing protease and phosphatase inhibitors. Determine protein concentration using the BCA assay kit. Equal volumes of protein from different groups were electrophoresed on sodium dodecyl sulfate-polyacrylamide gel (SDS-PAGE) and transferred to polyvinylidene difluoride (PVDF) membranes. Blocked with 5% skim milk in PBS for 2 h, then incubated overnight at 4 °C with the following antibodies: TLR4 (1:1000), MyD88 (1:1000), NF-κB (1:1000), p-NF-κB (1:1000), PSD95 (1:6000), GAP43 (1:1000), NeuN (1:2000), Bax (1:1000), Bcl-2 (1:1000), TNF-α (1:1000), IL-1β (1:1000), IL-6 (1:1000), Iba-1 (1:1000), CD206 (1:1000), iNOS (1:1000), GFAP (1:1000), p-Tau (1:1000), APP (1:1000) and p-APP (1:1000). Secondary antibodies from the same source as the primary antibodies were incubated at room temperature for 2 h. After thorough washing, protein bands were captured using the ChemiScope 6200 chemiluminescence imaging system (Shanghai Qinxiang Scientific Instruments Co., Ltd., Shanghai, China), followed by grayscale image analysis with ImageJ software.

### 2.14 Statistical analysis

All quantitative results are expressed as mean ± standard deviation (SD) and analyzed for statistical differences using SPSS 27.0 software. When data sets showed no significant deviation from normal distribution after normality testing and homogeneity of variance testing indicated consistent variance (*p* > 0.05), Tukey’s *post hoc* tests were performed. If homogeneity of variance testing indicated inconsistent variance (*p* < 0.05), Dunnett T3 *post-hoc* comparisons were conducted. *p* < 0.05 was considered statistically significant.

## 3 Result

### 3.1 Effect of 3–047 combined ICT on blood glucose and body weight in DACD mice

To observe the effect of drug combination on blood glucose and body weight of mice, we recorded blood glucose and body weight every 2 weeks. Blood glucose of db/db mice was significantly higher than that of db/m mice (*p* < 0.001). However, after 16 weeks of co-administration of the drugs, blood glucose of mice was significantly reduced (*p* < 0.01, *p* < 0.05), and blood glucose of the Met group showed a significant trend of reduction (*p* < 0.001) (**Figure 1C**); the administration did not affect the body weight of db/db mice (**Figure 1D**). To assess homeostatic model assessment of insulin resistance in diabetic mice, we measured fasting blood glucose as well as fasting insulin levels. db/db mice showed a significant increase in fasting blood glucose compared to db/m mice (*p* < 0.01), and blood glucose was significantly improved after drug administration (*p* < 0.01, *p* < 0.05) (**Figure 1E**), but there was no significant difference in insulin levels (**Figure 1F**), and the HOMA-IR showed a trend of difference identical to that of the fasting blood glucose with the same trend of difference (**Figure 1G**). That is, our results suggest that 3–047 combined with ICT can effectively improve blood glucose and insulin resistance in db/db mice.

### 3.2 3–047 combined ICT improves cognitive dysfunction in DACD mice

**Figure 2:**
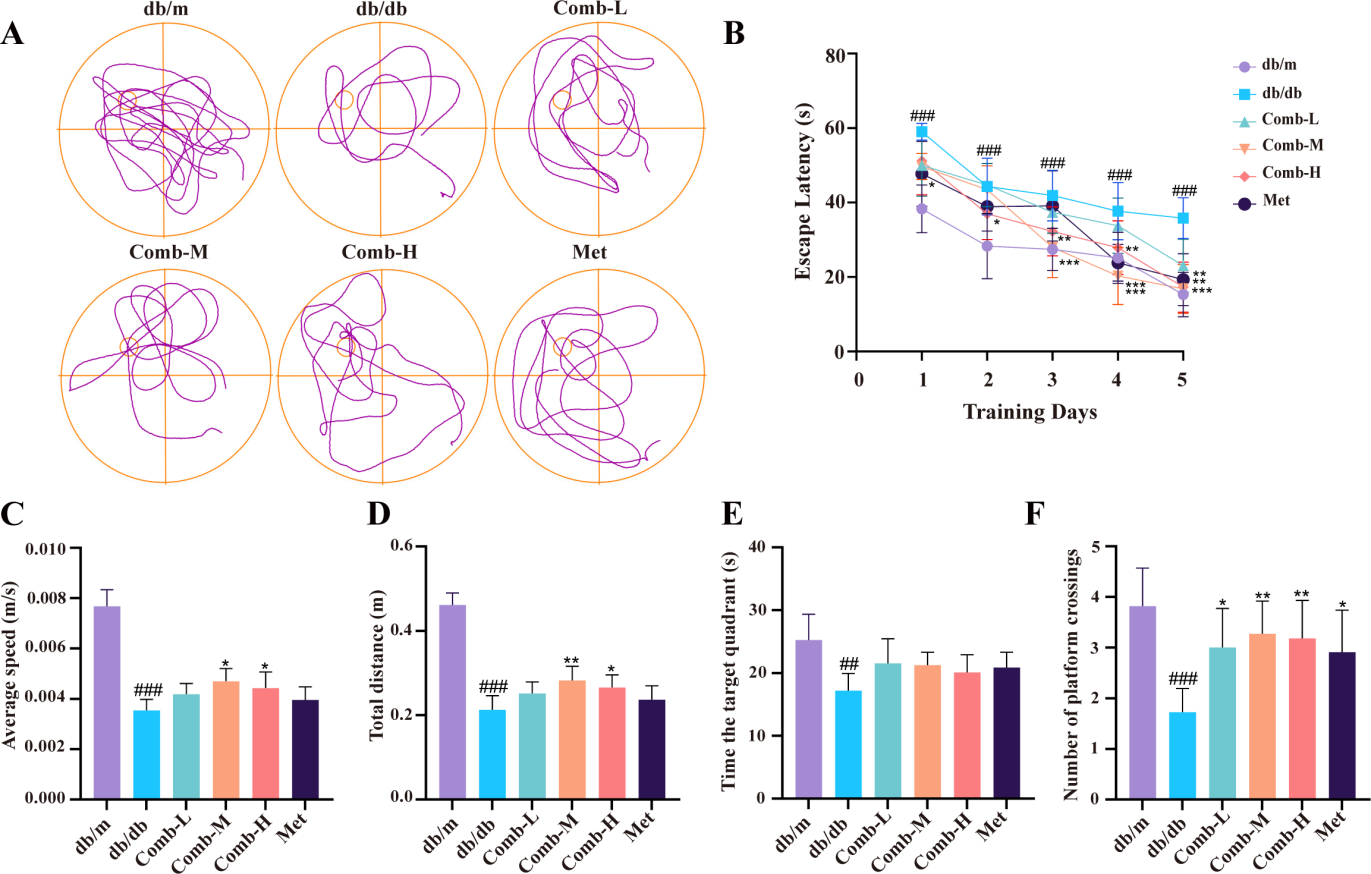
3–047 combined with ICT ameliorates cognitive dysfunction in DACD mice. **(A)** Representative swimming trajectories in the MWM. **(B)** Latency to find the platform in the MWM. **(C)** Average swimming speed of mice. **(D)** Total swimming distance of mice. **(E)** Time spent in the platform quadrant by mice. **(F)** Number of platform crossings in MWM mice. Data are presented as mean ± SD (n = 11 per group). Compared with db/m mice, ^###^*p* < 0.001, ^##^*p* < 0.01; compared with db/db mice, ^***^p < 0.001, ^**^p < 0.01, ^*^p < 0.05.

The spatial learning memory ability of db/db mice was observed by MWM. During the 5-day hidden platform training phase, the latency of mice to reach the platform gradually decreased, but the latency of db/db mice to reach the platform was still significantly longer than that of db/m mice (*p* < 0.001), and the latency was significantly reduced after the drug administration intervention (*p* < 0.05, *p* < 0.01, *p* < 0.001) (**Figure 2B**). After the platform was removed, any adverse effects of the drug were observed by swimming speed as well as the total distance swum, and the spatial exploration memory ability of the mice was observed by recording the number of times the mice traversed the platform and the time spent in the quadrant where the platform was located. Compared with db/m mice, db/db mice showed a significant decrease in swimming speed (*p* < 0.001) and total distance traveled for exploration (*p* < 0.001). After treatment, there was a significant increase in speed in the combined medium and high-dose groups (*p* < 0.05), and the total distance traveled in the combined medium as well as the combined high-dose was significantly increased (*p* < 0.01, *p* < 0.05) (**Figures 2C, D**), indicating that combined drug therapy effectively enhances aquatic locomotor ability in mice. Additionally, db/db mice exhibited significantly reduced platform crossings and time spent in the platform quadrant (*p* < 0.01, *p* < 0.05) (**Figures 2E, F**), and there was no significant difference in the time spent in the quadrant where the platform was located after treatment with the drug, but the number of times mice traversed the platform was significantly increased (*p* < 0.01, *p* < 0.05). These results suggest that 3–047 combined with ICT significantly improved cognitive dysfunction in diabetic mice.

### 3.3 3–047 combined ICT reverses neuronal damage in DACD mice

**Figure 3:**
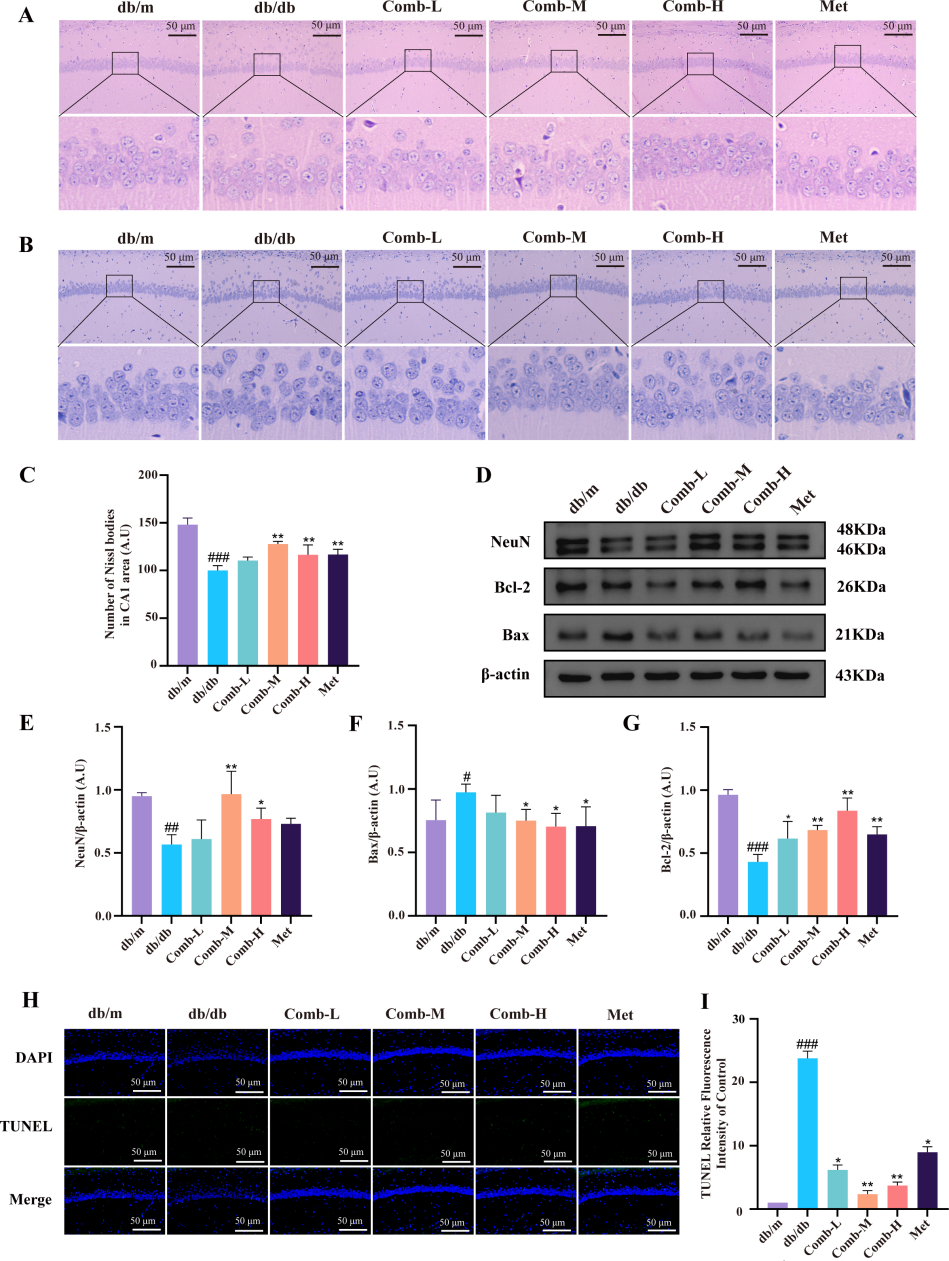
3–047 combined with ICT attenuates neuronal apoptosis and histological damage in DACD mice. **(A)** Representative H&E staining and magnified images images of the hippocampal CA1 region. Scale bar = 50 μm (200×). **(B)** Representative Nissl staining and magnified images of the hippocampal CA1 region. Scale bar = 50 μm (200×). **(C)** Quantitative analysis of Nissl staining. **(D)** Representative western blot images of NeuN, Bax, Bcl-2, and β-actin in hippocampal tissue. **(E-G)** Quantitative analysis of NeuN, Bax and Bcl-2 protein expression levels relative to β-actin in each group. **(H)** Representative TUNEL staining images of the hippocampal CA1 region, scale bar = 50 μm (200×). **(I)** Quantitative statistics of the fluorescence intensity of TUNEL staining relative to the control group. Data are presented as mean ± SD (n = 3 per group). Compared with db/m mice, ^###^*p* < 0.001, ^##^*p* < 0.01, ^#^*p* < 0.05; compared with db/db mice, ^**^p < 0.01, ^*^p < 0.05.

Cognitive impairment is associated with neuronal damage (29). We observed disordered and dispersed neuronal alignment in db/db mice through H&E staining (**Figure 3A**). Concurrently, Nissl staining demonstrated reduced and dispersed Nissl bodies (*p* < 0.05) (**Figures 3B, C**), reflecting neuronal apoptosis and dispersion. TUNEL staining further validated these findings (**Figures 3H, I**), confirming increased apoptotic neurons and nuclear condensation in db/db mice, which was significantly alleviated following combination treatment. In addition, we detected the expression of NeuN protein (an indicator of neuronal survival) using Western blotting. The expression of NeuN was significantly downregulated in db/db mice (*p* < 0.05) but was significantly restored in the combined medium- and high-dose groups (*p* < 0.01, *p* < 0.05) (**Figure 3E**). Furthermore, we examined the expression of pro-apoptotic protein Bax and anti-apoptotic protein Bcl-2. db/db mice showed a significant increase in the expression of Bax (*p* < 0.05) and a significant decrease in the expression of Bcl-2 (*p* < 0.001). Conversely, both the medium- and high-dose groups showed a significant decrease in Bax expression (*p* < 0.05), whereas Bcl-2 expression increased across all treated groups (*p* < 0.01, *p* < 0.05) (**Figures 3F, G**). These results indicate that neurons in mice with diabetic cognitive dysfunction exhibit shrinkage and apoptosis, which can be alleviated by combined drug treatment.

### 3.4 3–047 combined ICT improves hippocampal ultrastructure in DACD mice

**Figure 4:**
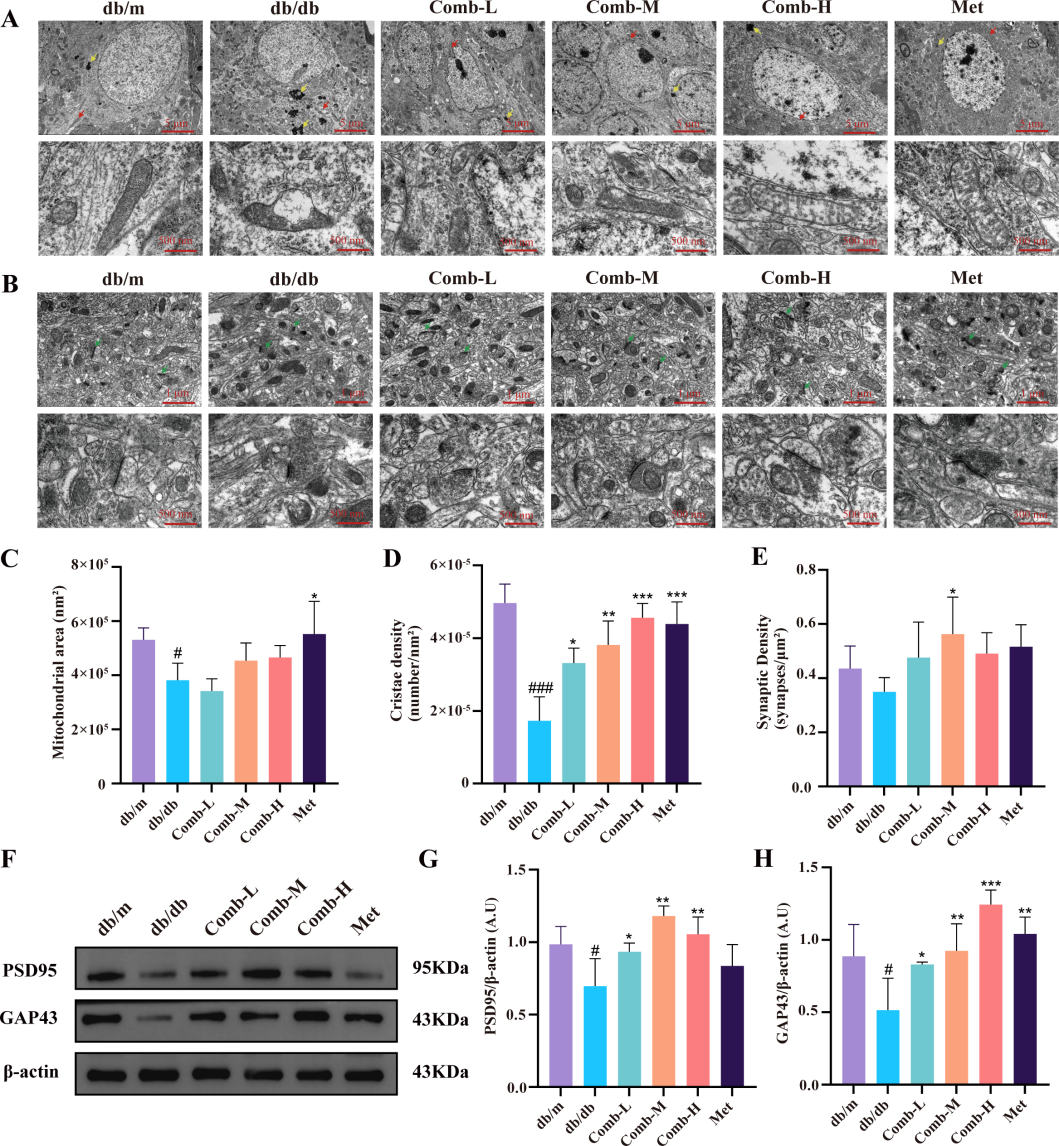
3–047 combined with ICT ameliorates hippocampal mitochondrial and synaptic ultrastructural defects in DACD mice. **(A)** Representative ultrastructure images of hippocampal mitochondria and lysosomes. Red arrows: mitochondria; yellow arrows: lysosomes. Scale bar = 5 μm (3k×), 500 nm (25k×). **(B)** Representative ultrastructure images of hippocampal synapses. Green arrow: synapse. Scale bar = 1 μm (10k×), 500 nm (25k×). **(C)** Quantitative analysis of mitochondrial area. **(D)** Quantitative analysis of cristae density in each mitochondrion. **(E)** Quantitative analysis of synaptic density. **(F)** Representative western blot images of PSD95, GAP43, and β-actin in hippocampal tissue. **(G-H)** Quantitative analysis of PSD95 and GAP43 protein expression levels relative to β-actin in each group. Data are presented as mean ± SD (n = 3 per group; 3 regions per mouse were analyzed for mitochondria and synapses). Compared with db/m mice, ^#^*p* < 0.05; compared with db/db mice, ^***^p < 0.001, ^**^p < 0.01, ^*^p < 0.05.

The ultrastructure of the hippocampus was assessed by TEM. We found that compared to db/m mice, mitochondria in the hippocampal tissue of db/db mice exhibited characteristics such as swelling, rupture, cristae disruption, and disappearance (*p* < 0.01, *p* < 0.05). Following drug treatment, mitochondrial vacuolation was alleviated and cristae density was significantly increased (*p* < 0.001, *p* < 0.01, *p* < 0.05). Although the increase in mitochondrial area did not reach statistical significance, a clear recovery trend was observed (**Figures 4A, C, D**). Simultaneously, we found that while synaptic density in db/db mice did not decrease significantly compared to db/m mice, synaptic structure was markedly impaired, manifested by widened synaptic clefts, narrowed postsynaptic dense regions (**Figures 4B, E**). Following drug treatment, hippocampal synaptic clefts did not show marked widening, synaptic vesicles were abundant. Interestingly, synaptic density significantly increased at the medium dose (*p* < 0.05). Further analysis of the postsynaptic marker PSD95 and the synaptic plasticity marker GAP43 revealed that compared to db/m mice, db/db mice exhibited significantly reduced PSD95 expression (*p* < 0.05) (**Figure 4G**) and GAP43 expression (*p* < 0.05) (**Figure 4H**). Following drug intervention, PSD95 and GAP43 expression significantly increased (*p* < 0.001, *p* < 0.01, *p* < 0.05). Collectively, these findings indicate that combined drug therapy exerts a protective effect on hippocampal ultrastructure in diabetic mice.

### 3.5 3–047 combined ICT ameliorates neuronal degeneration, p-Tau abnormalities, and amyloid deposition in DACD mice

**Figure 5:**
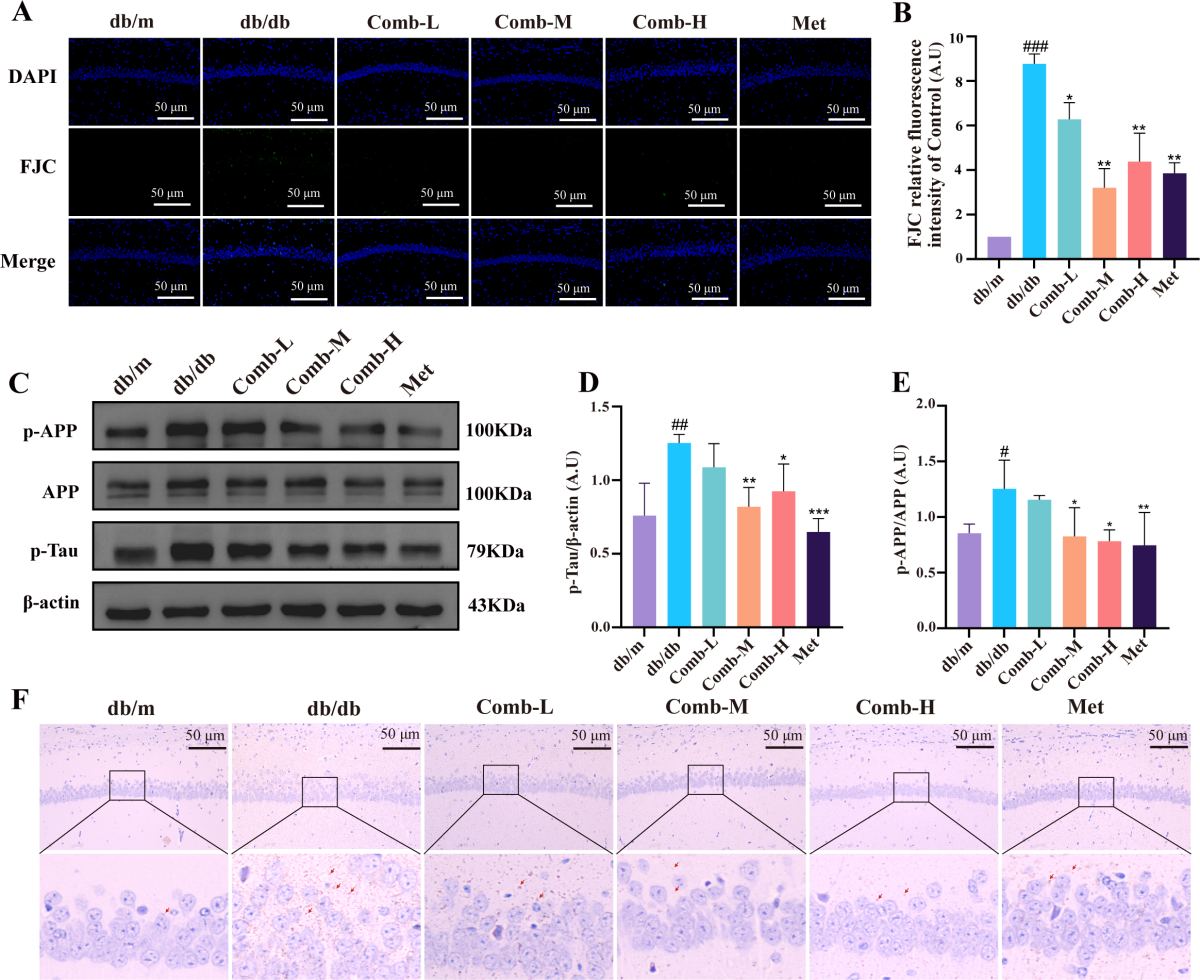
3–047 combined ICT ameliorates P-Tau abnormalities, amyloid deposition, and neuronal degeneration in DACD mice. **(A)** Representative FJC staining images of the hippocampal CA1 region, scale bar = 50 μm (200×). **(B)** Quantitative statistics of FJC staining fluorescence intensity relative to the control group. **(C)** Representative western blot images of p-APP, APP, p-Tau, and β-actin in hippocampal tissue. **(D, E)** Quantitative analysis of the p-APP/APP ratio, and p-Tau protein expression levels relative to β-actin. **(F)** Representative Congo red staining and magnified images of the hippocampal CA1 region, scale bar = 50 μm (200×), red arrows indicate amyloid deposits. Data are presented as mean ± SD (n = 3 per group). Compared with db/m mice, ^###^*p* < 0.001, ^##^*p* < 0.01, ^#^*p* < 0.05; compared with db/db mice, ^***^p < 0.001, ^**^p < 0.01, ^*^p < 0.05.

We observed the degeneration of neurons in mice by FJC staining. The fluorescence intensity of degenerated neurons was significantly enhanced in db/db mice compared with db/m mice (*p* < 0.001) (**Figures 5A, B**), suggesting that degeneration of some neurons existed in DACD mice. However, pharmacological treatment effectively reversed neuronal degeneration in DACD mice (*p* < 0.01, *p* < 0.05).

In addition, we found that the expression of tau protein phosphorylation (p-Tau) was increased in db/db mice compared with db/m mice (*p* < 0.01) (**Figure 5D**), whereas p-Tau levels were significantly decreased after the treatment (*p* < 0.01, *p* < 0.05). Similarly, a significant elevation in p-APP protein expression was observed (*p* < 0.05) (**Figure 5E**), indicating aberrant APP processing. However, p-APP expression was significantly reduced after drug treatment (*p* < 0.001, *p* < 0.01, *p* < 0.05). We also observed significant amyloid plaques in the hippocampal region of db/db mice using Congo red staining, and the deposition of amyloid plaques in the hippocampal region was significantly reduced after drug treatment (**Figure 5F**).

### 3.6 3–047 combined ICT alleviates neuroinflammation in DACD mice

**Figure 6:**
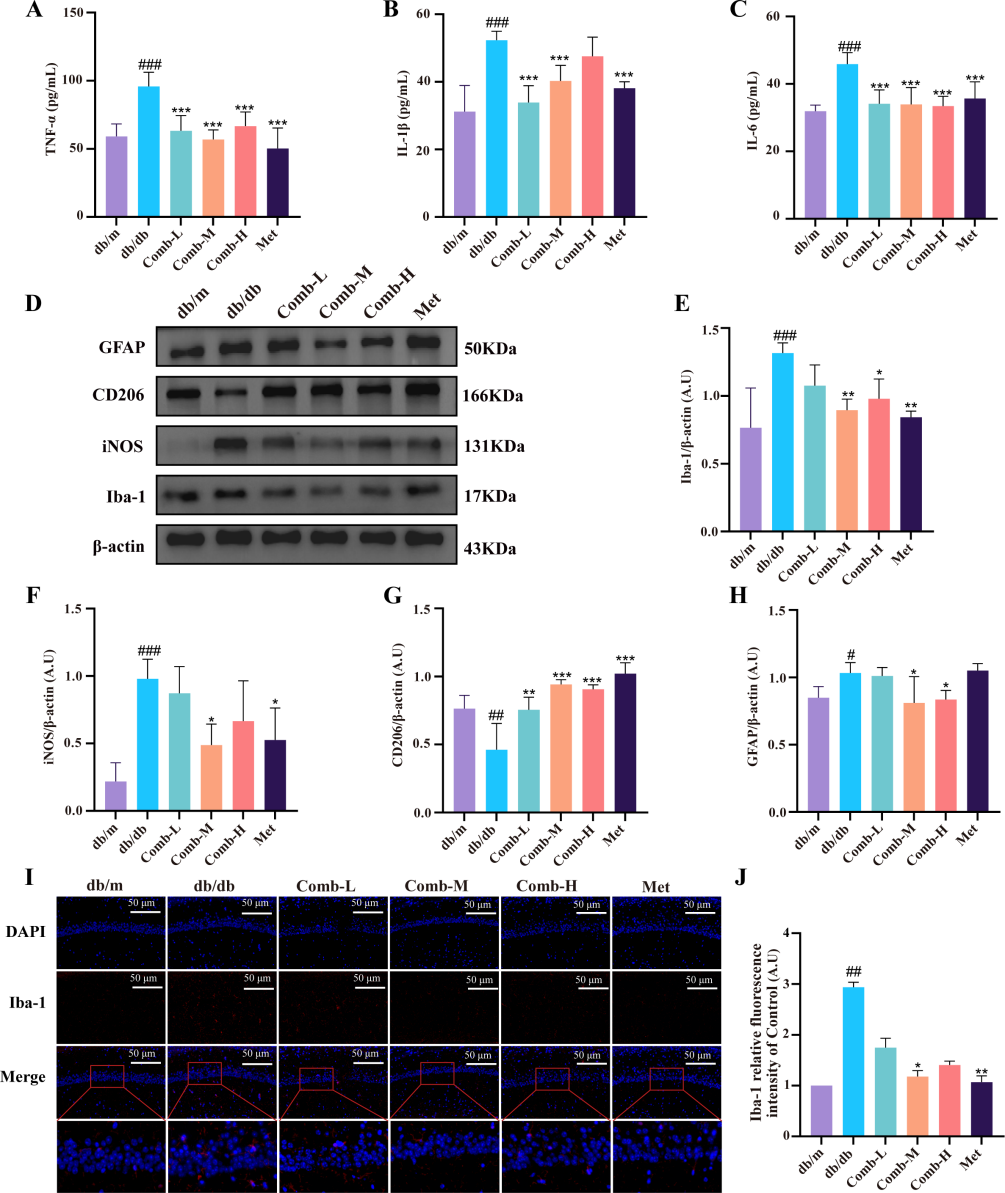
3–047 combined with ICT alleviates neuroinflammation in DACD mice. **(A–C)** ELISA detection of TNF-α, IL-1β, and IL-6 levels in mouse serum. **(D)** Representative Western blot images of Iba-1, iNOS, CD206, GFAP, and β-actin in hippocampal tissue. **(E–H)** Quantitative analysis of Iba-1, iNOS, CD206, and GFAP protein expression levels relative to β-actin in each group. **(I)** Representative Iba-1 immunofluorescence and magnified images of the hippocampal CA1 region, scale bar = 50 μm (200×). **(J)** Normalized quantitative statistics of Iba-1 immunofluorescence intensity relative to the control group. Data are presented as mean ± SD. [n = 6 per group for **(A-C)**; n = 3 per group for **(D-J)**]. Compared with db/m mice, ^###^*p* < 0.001, ^##^*p* < 0.01, ^#^*p* < 0.05; compared with db/db mice, ^***^p < 0.001, ^**^p < 0.01, ^*^p < 0.05.

Neuroinflammation is one of the key factors contributing to cognitive impairment in diabetes (14). First, we measured serum levels of proinflammatory cytokines including TNF-α, IL-1β, and IL-6. We found that db/db mice exhibited significantly higher expression levels of TNF-α, IL-1β, and IL-6 compared to db/m mice (*p* < 0.001) (**Figures 6A-C**). Following drug treatment, the levels of these pro-inflammatory factors decreased markedly (*p* < 0.001), though IL-1β levels in the high-dose combination group did not show a significant reduction. Subsequently, we performed Western blot analysis for the microglial marker Ionized calcium-binding adapter molecule 1 (Iba-1) and the astrocytic marker GFAP to preliminarily assess the development of neuroinflammation in the mice (**Figure 6D**). Iba-1 and GFAP expression was markedly elevated in db/db mice (*p* < 0.001, *p* < 0.05) (**Figures 6E, H**), and both were significantly reduced after drug treatment (*p* < 0.01, *p* < 0.05). Concurrently, Iba-1 immunofluorescence revealed significantly higher fluorescence intensity in db/db mice compared to db/m mice (p < 0.01), with a marked reduction post-treatment (*p* < 0.01, *p* < 0.05) (**Figures 6I-J**). Next, we examined microglial phenotype expression. db/db mice exhibited significantly elevated expression of the M1 marker iNOS (*p* < 0.001) (**Figure 6F**) and markedly reduced expression of the M2 marker CD206 (*p* < 0.01) (**Figure 6G**). This indicates that the high-glucose environment *in vivo* activated the pro-inflammatory phenotype of hippocampal microglia while suppressing the anti-inflammatory phenotype. In response to the combined therapy, the expression levels of M1/M2 markers were significantly reversed (*p* < 0.001, *p* < 0.01, *p* < 0.05). These results demonstrate that combination treatment can markedly alleviate the progression of neuroinflammation in diabetic mice.

### 3.7 3–047 combined ICT inhibits TLR4/MyD88/NF-κB pathway in DACD mice

**Figure 7:**
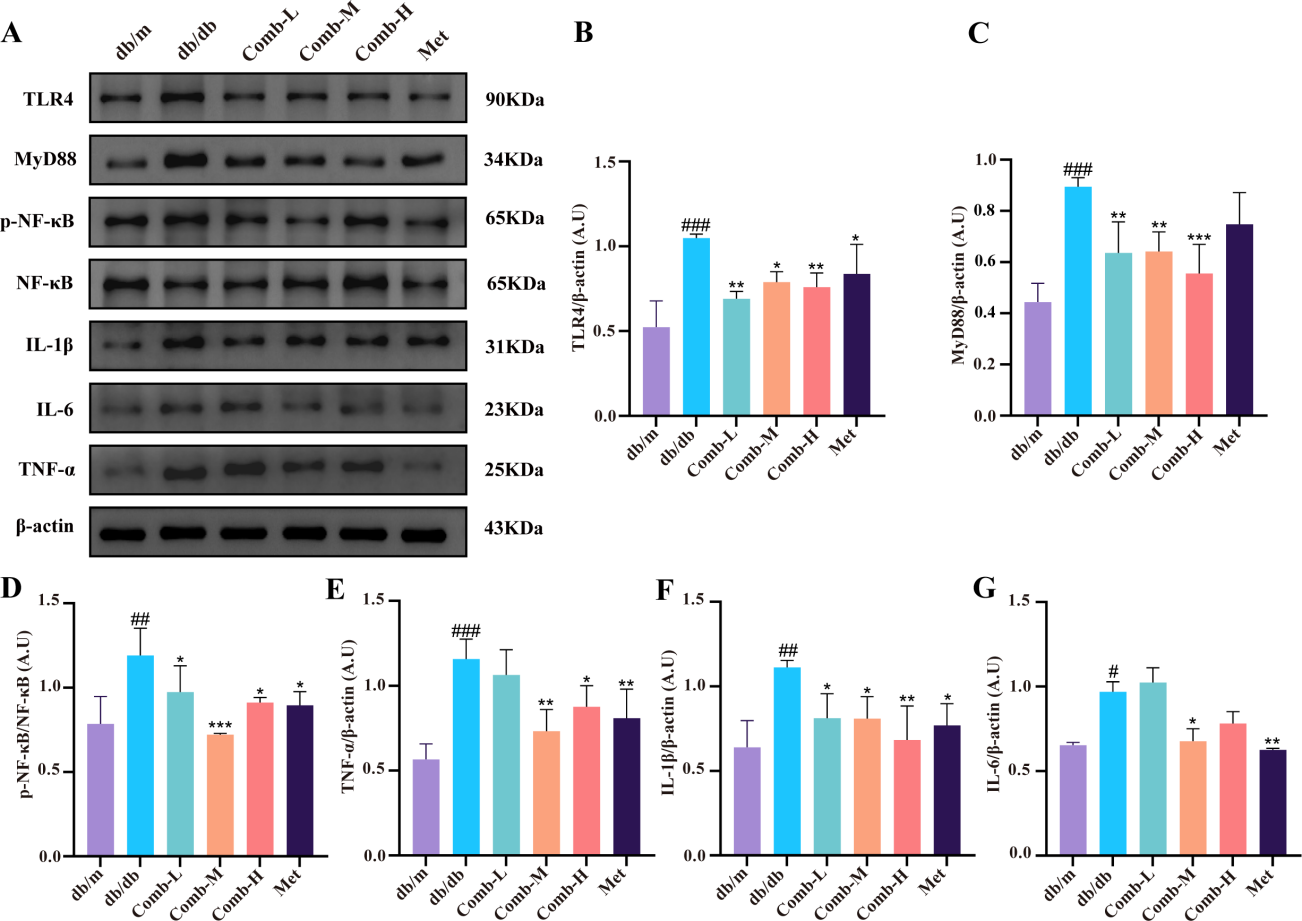
3–047 combined with ICT inhibits the TLR4/MyD88/NF-κB signaling pathway in DACD mice. **(A)** Representative Western blot images of TLR4, MyD88, p-NF-κB, NF-κB, IL-1β, IL-6, TNF-α, and β-actin in hippocampal tissue. **(B–G)** Quantitative analysis of TLR4, MyD88, IL-1β, IL-6, and TNF-α protein expression levels relative to β-actin, and the p-NF-κB/NF-κB ratio. Data are presented as mean ± SD (n = 3 per group). Compared with db/m mice, ^###^*p* < 0.001, ^##^*p* < 0.01, ^#^*p* < 0.05; compared with db/db mice, ^***^p < 0.001, ^**^p < 0.01, ^*^p < 0.05.

We hypothesize that the combined treatment of 3–047 and ICT may alleviate neuroinflammation, hippocampal ultrastructural damage, and neuronal apoptosis in DACD through the TLR4/MyD88/NF-κB pathway, so we analyzed the expression of key proteins in this signaling pathway. Western blot analysis revealed significantly higher TLR4 expression in db/db mice compared to db/m mic (*p* < 0.001) (**Figure 7B**), along with elevated MyD88 expression (*p* < 0.001) (**Figure 7C**), and markedly increased p-NF-κB expression (*p* < 0.01) (**Figure 7D**). Following combined drug treatment, TLR4/MyD88/NF-κB protein expression levels were significantly reduced (*p* < 0.001, *p* < 0.01, *p* < 0.05). Furthermore, compared with db/m mice, db/db mice exhibited markedly elevated protein expression levels of inflammatory mediators TNF-α, IL-1β, and IL-6 in hippocampal tissue (*p* < 0.001, *p* < 0.01, *p* < 0.05) (**Figures 7E-G**), with a downward trend in expression following treatment (*p* < 0.01, *p* < 0.05). These findings indicate that the combined 3–047 and ICT therapy likely exerts its effects on DACD through the TLR4/MyD88/NF-κB pathway.

## 4 Discussion

DACD has gradually emerged as one of the major complications of diabetes. The db/db mouse is a spontaneous type 2 diabetes model derived from C57 BLKS mice with a mutation in the *Lepr* gene. It exhibits characteristic features including insulin resistance, persistent hyperglycemia, and obesity. Multiple studies have demonstrated significant cognitive dysfunction in db/db mice (6, 30, 31). This study employed db/db mice as experimental subjects to evaluate the effects of 3–047 combined with ICT on cognitive impairment in diabetes. Results demonstrated that the combined drug therapy significantly improved cognitive dysfunction in db/db mice.

The MWM test was used for preliminary assessment of cognitive impairment in DACD (32). Compared with db/m mice, db/db mice exhibited prolonged escape latency and fewer platform crossings, indicating impaired cognitive function. This finding aligns with previous reports (33). We observed that db/db mice exhibited significantly reduced swimming velocity and total distance traveled during the spatial memory exploration phase compared to db/m mice. Following combined drug treatment, mice demonstrated significant improvements in training latency, swimming velocity, and total distance traveled. This outcome may be influenced by the mice’s metabolic state. However, spatial memory assessment in DACD mice relies more heavily on platform crossings. Mice in the 3–047 combined with ICT treatment group exhibited significantly more platform crossings than db/db mice, indicating that this combination therapy improved spatial memory in DACD mice. These behavioral deficits are closely linked to molecular pathology in the hippocampus. Previous studies have demonstrated that mice with DACD exhibit hyperphosphorylated tau protein and Aβ deposits in the hippocampus (34). Hyperphosphorylated tau readily forms oligomers. These oligomers not only induce inflammatory factors but also lead to synaptic dysfunction and loss, further accelerating the progression of neurodegenerative diseases (35). Simultaneously, APP phosphorylation increases Aβ production, leading to the formation of amyloid plaques (36). Aβ also exacerbates tau phosphorylation, accelerating the progression of DACD (37). Neuronal cell body integrity influences cognitive impairment progression. Yu et al. observed marked neuronal cell body degeneration in diabetic cognitive impairment mice (38). In this study, we observed that following drug treatment, db/db mice exhibited significantly reduced expression of p-Tau and p-APP in hippocampal tissue, markedly improved amyloid deposition, and a substantial decrease in degenerating neurons. These findings suggest that controlling amyloid deposition and tau phosphorylation may represent effective therapeutic strategies for DACD.

Under the influence of prolonged hyperglycemia, hippocampal neurons in diabetic mice exhibit shrinkage and a dispersed arrangement (29). Excessive phosphorylation of tau protein induces neuronal apoptosis (39), further exacerbating the progression of cognitive impairment. In this study, H&E staining and Nissl staining revealed that after drug treatment, hippocampal neurons in mice exhibited compact arrangement, and the number of Nissl bodies was markedly higher than in db/db mice. To further evaluate the extent of cell death, we performed TUNEL staining. The results indicated that compared with db/db mice, the TUNEL fluorescence intensity in the hippocampal region of mice after drug intervention was significantly reduced. Consistent with these histological findings, detection of protein expression levels for NeuN, Bax, and Bcl-2 indicated that the combined intervention significantly attenuated neuronal apoptosis in mice. When DACD occurs, it is often accompanied by damage to mitochondrial ultrastructure in the hippocampal region. This damage may lead to impaired energy metabolism in hippocampal neurons, thereby affecting normal cellular physiological activities (32, 40). Disruption of hippocampal synaptic structures frequently occurs concurrently. Synapses serve as critical sites for neural signal transmission, and their structural loss impedes information exchange between neurons (41, 42). Our study demonstrates that the combination of 3–047 and ICT alleviates mitochondrial ultrastructural abnormalities in hippocampal tissue and improves synaptic structural integrity in db/db mice. This finding was further validated by assessing the expression of PSD95 and GAP43 proteins. Considering the behavioral indicators discussed earlier, we hypothesize that mitochondrial damage may co-occur with behavioral deficits, a finding consistent with previous studies.

Diabetes can induce systemic low-grade inflammation, leading to the production of pro-inflammatory cytokines including TNF-α, IL-1β, and IL-6, thereby exacerbating neuroinflammation and promoting cognitive dysfunction (43). Microglia and astrocytes are key regulators of the immune response and inflammation. The progression of neuroinflammation can be preliminarily observed by detecting the microglia marker Iba-1 and the astrocyte marker GFAP (44). When stimulated by cell receptors such as Toll-like receptors (TLRs) or nuclear oligomerization domain-like receptors, microglia produce pro-inflammatory factors including TNF-α, IL-1β, IL-6, nitric oxide, and proteases all closely associated with the progression of neurodegenerative diseases (12, 45). In addition to their pro-inflammatory potential and impact on blood-brain barrier function (46), astrocytes also exert detrimental effects on neurodegenerative diseases. This study focused on observing microglial changes and more accurately assessing neuroinflammation progression by detecting the expression levels of M1 and M2 microglial markers. Results indicate that db/db mice exhibit significant neuroinflammation. Co-administration of 3–047 with ICT markedly reduced Iba-1 and GFAP expression, enhancing anti-inflammatory effects and effectively suppressing inflammatory responses. However, due to limited animal numbers, this study could not dynamically monitor the M1/M2 polarization process of microglia.

The TLR4/MyD88/NF-κB pathway, as a key regulatory axis of innate immune responses, can rapidly respond to signals related to metabolic disorders and inflammation within the central nervous system (47, 48). This response influences the progression of cognitive dysfunction. Studies indicate that inhibiting the TLR4/MyD88/NF-κB signaling pathway effectively alleviates Alzheimer’s disease symptoms (48). Fan et al. also found that suppressing this pathway significantly mitigates cognitive impairment in KK-ay mice with type 2 diabetes (49). Through Western blot analysis, we observed significantly elevated expression of TLR4/MyD88/NF-κB pathway-related proteins in db/db mice, indicating the activation of this axis in DACD. The TLR4 signaling pathway is closely associated with IDO-1-mediated metabolic disorders. Activation of the TLR4/NF-κB-related pathway induces IDO overexpression (50). Studies indicate that TLR4/NF-κB activation induces IDO-1 expression, increasing tryptophan catabolism via the kynurenine pathway. This leads to heightened synthesis of downstream neurotoxic metabolites (e.g., quinolinic acid), triggering excitotoxicity (51). Concurrently, evidence indicates that inhibiting IDO-1 expression effectively reduces levels of TLR4 pathway-associated proteins (52). As an IDO-1 inhibitor, we hypothesize that 3–047 may also effectively suppress the TLR4 pathway. Liu et al. found that ICT may exert neuroprotective effects by inhibiting lipopolysaccharide-induced TLR4-associated NF-κB signaling (53). In this study, following combined 3–047 and ICT intervention in db/db mice, expression of proteins associated with the TLR4/MyD88/NF-κB pathway was downregulated. This indicates that combined 3–047 and ICT therapy retains the ability to inhibit the TLR4/MyD88/NF-κB pathway. Furthermore, although reduced p-NF-κB levels were observed, additional functional analyses, such as nuclear translocation assays, DNA-binding activity assays, and transcriptional analyses are warranted to confirm NF-κB pathway inhibition.

This study demonstrates for the first time that the combination of 3–047 and ICT exhibits significant therapeutic efficacy for DACD. However, certain limitations remain. The study did not include monotherapy groups (3–047 alone or ICT alone). Although the combination therapy regimen showed overall superiority over the control group, the available data cannot determine whether the observed improvements stem from synergistic effects, additive interactions, or are primarily driven by a single component. Future studies incorporating monotherapy groups are needed to quantify synergistic effects. Due to factors such as obesity and reduced mobility in db/db mice, this study only performed the MWM test, limiting the assessment of the drugs’ broader impact on cognitive dysfunction. Further clarification of the mechanism and effects of 3–047 combined with ICT on DACD requires future studies utilizing multiple diabetic models and *in vitro* experiments.

## 5 Conclusion

Our results showed that 3–047 combined with ICT could effectively alleviate DACD pathology, reduce neuronal apoptosis, β-amyloid deposition and tau phosphorylation, enhance hippocampal synaptic plasticity, reduce neuroinflammation, and improve spatial learning and memory in diabetic mice. These results suggest that these changes may be related to the inhibition of TLR4/MyD88/NF-κB pathway. Therefore, 3–047 combined with ICT is expected to be an effective treatment for DACD.

## Data Availability

The original contributions presented in the study are included in the article/Supplementary Material. Further inquiries can be directed to the corresponding authors.
