# Supplementary material for: The novel IDO-1 inhibitor 3-047 combined with icaritin ameliorates neuroinflammation and diabetes-associated cognitive dysfunction with suppression of TLR4/MyD88/NF-κB signaling
Source: Front Immunol. 2026 Jan 26;17:1704307. doi: 10.3389/fimmu.2026.1704307 (PMC12883362; doi:10.3389/fimmu.2026.1704307)

Repeat 1

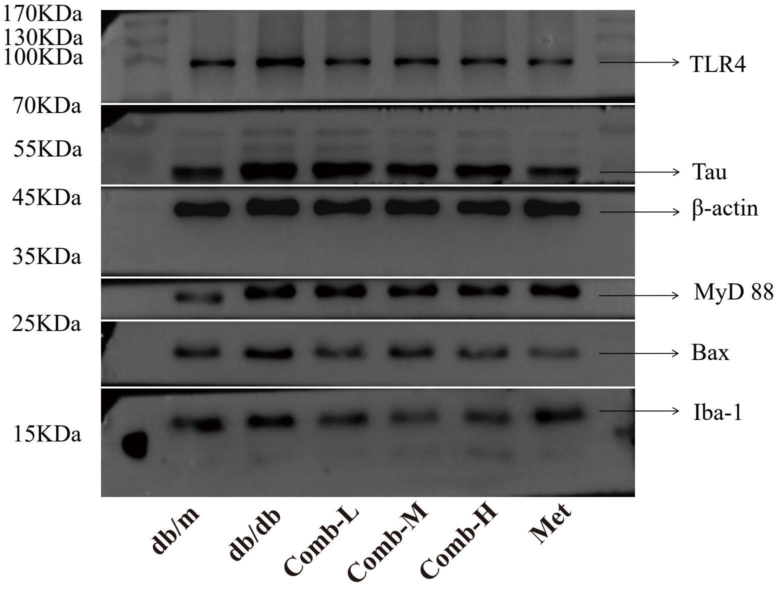

Repeat 2

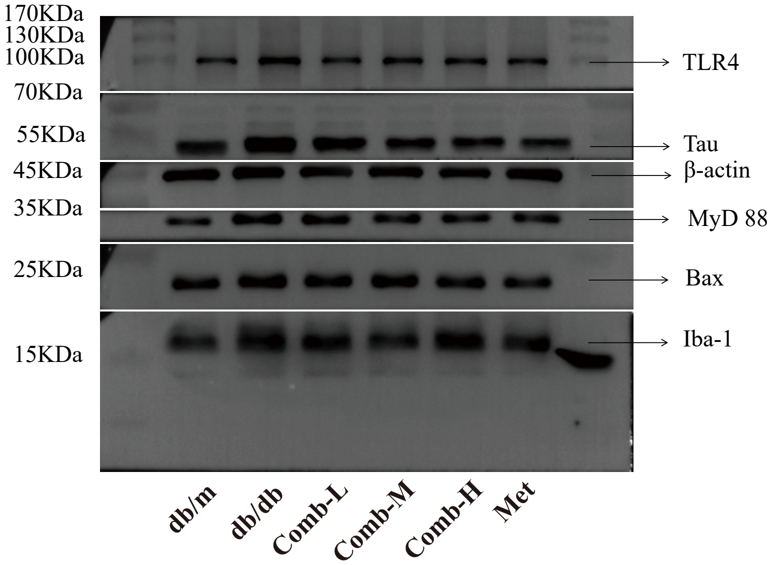

Repeat 3

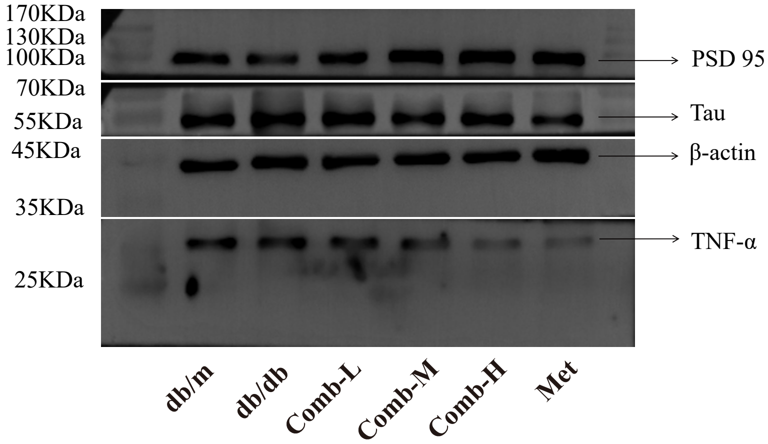

Repeat 4

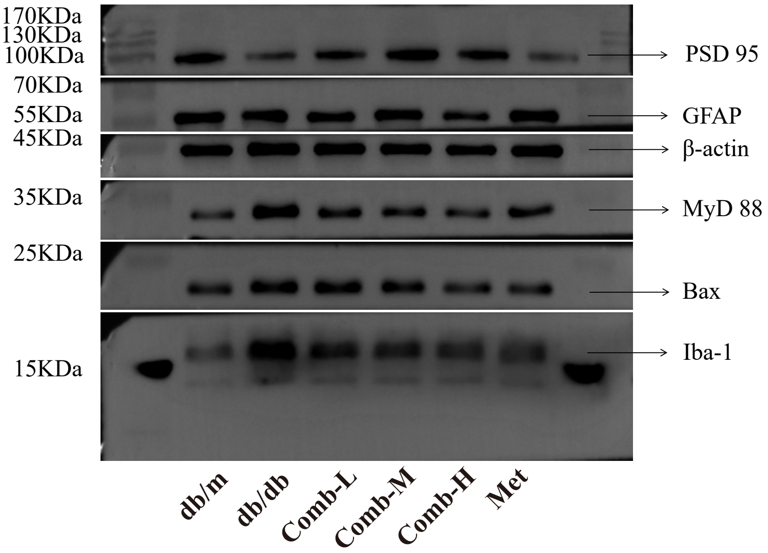

Repeat 5

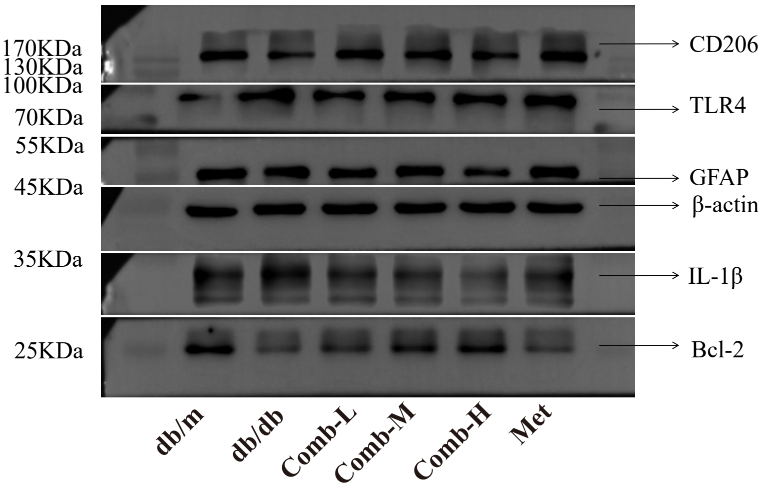

Repeat 6

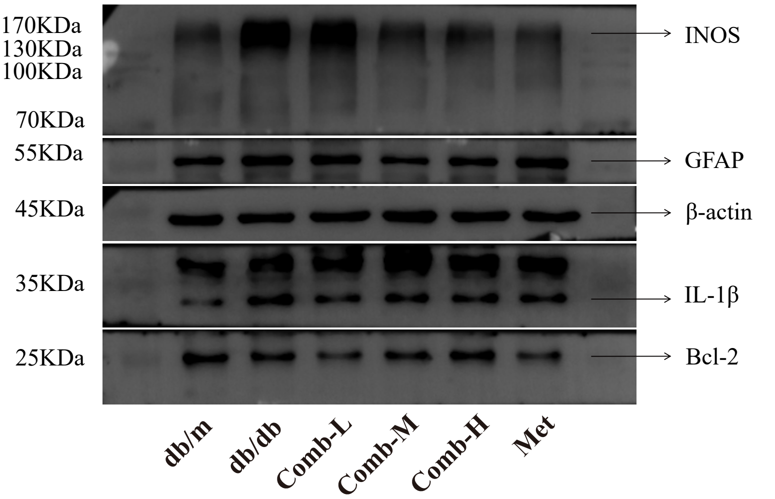

Repeat 7

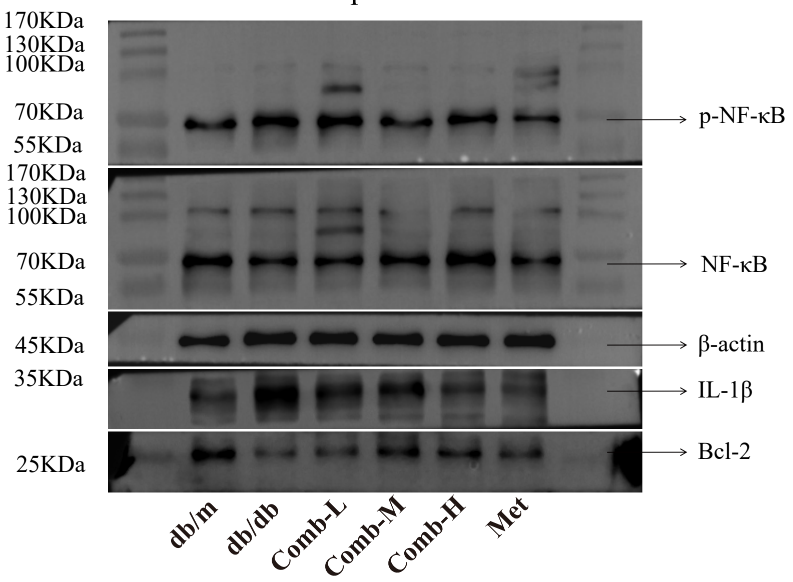

Repeat 8

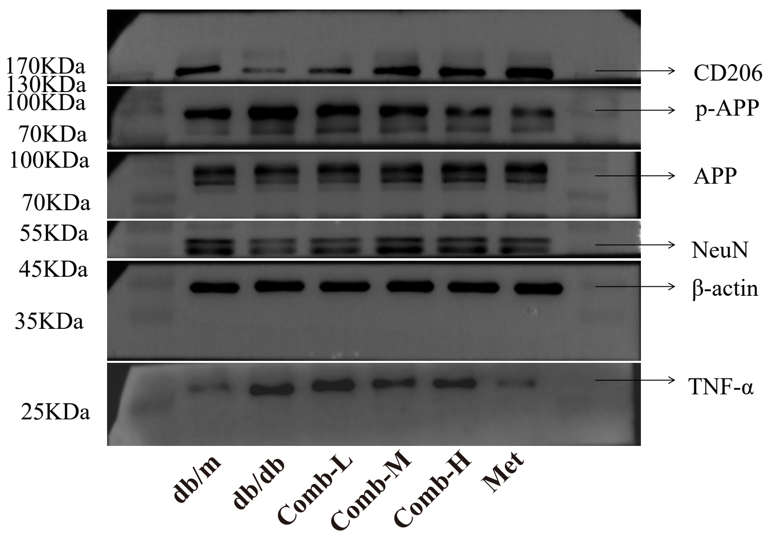

Repeat 9

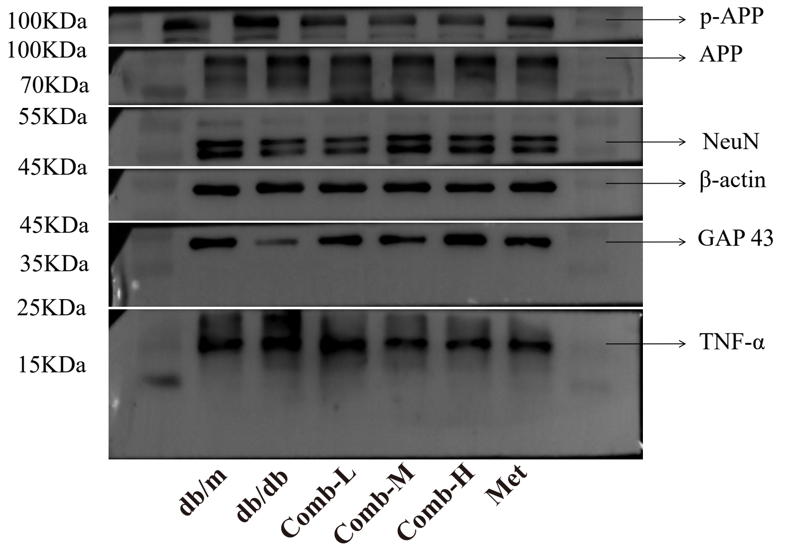

Repeat 10

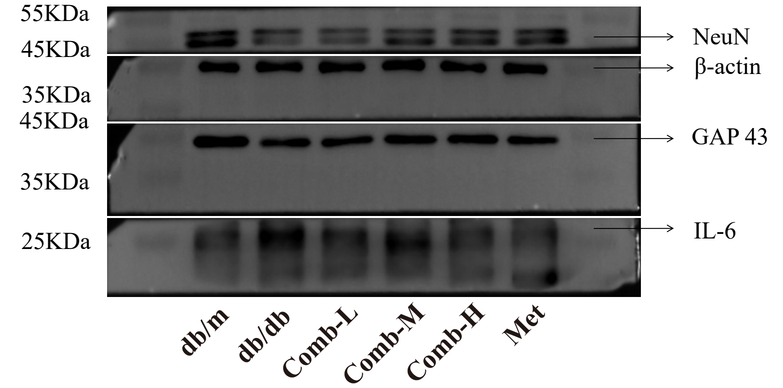

Repeat 11

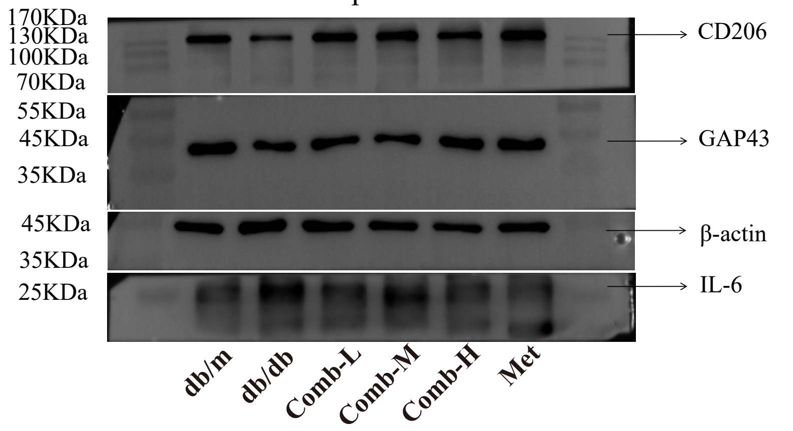

Repeat 12

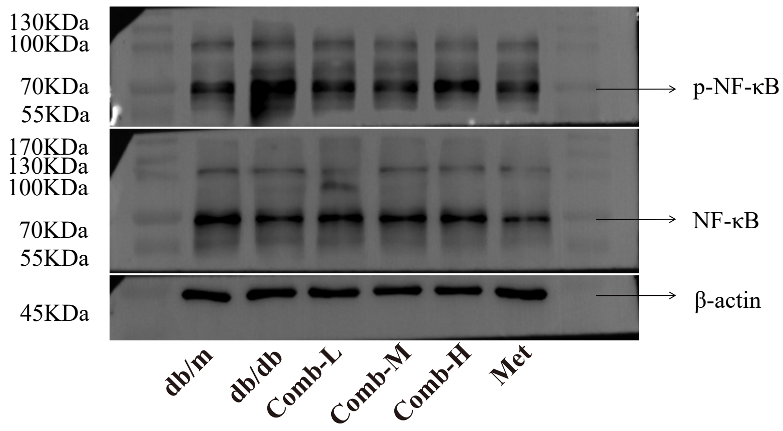

Repeat 13

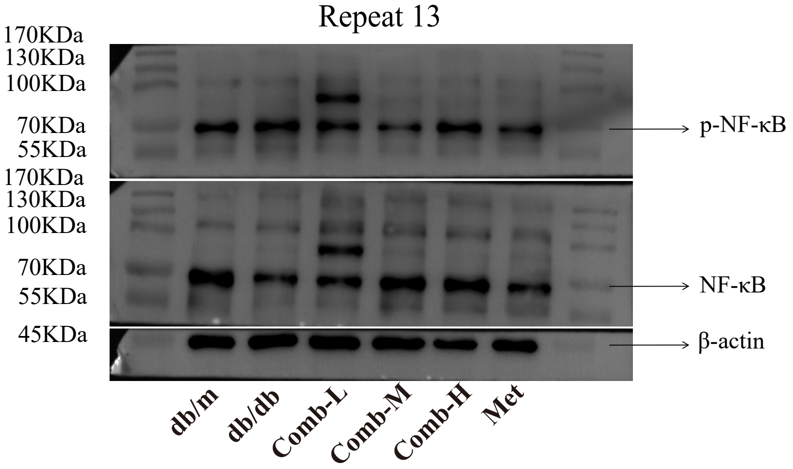

Repeat 14

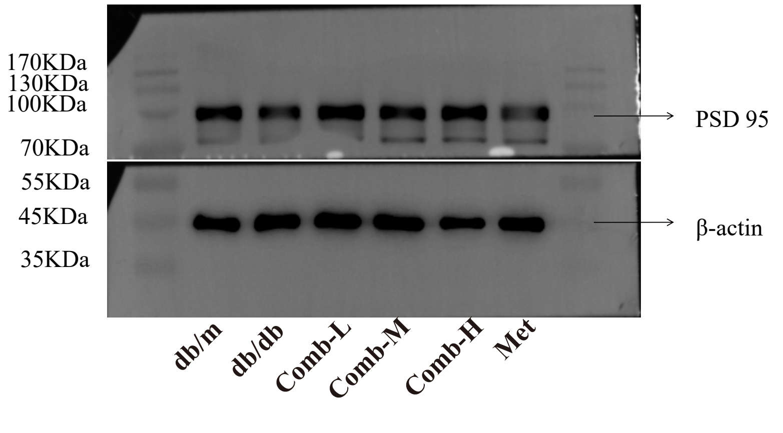

Repeat 15

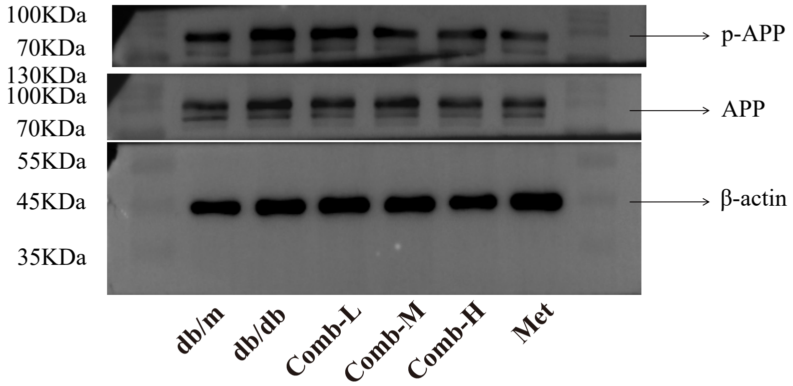

Repeat 16

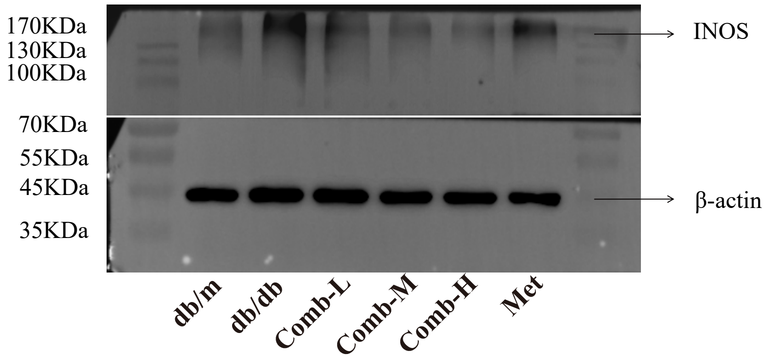

Figure 17

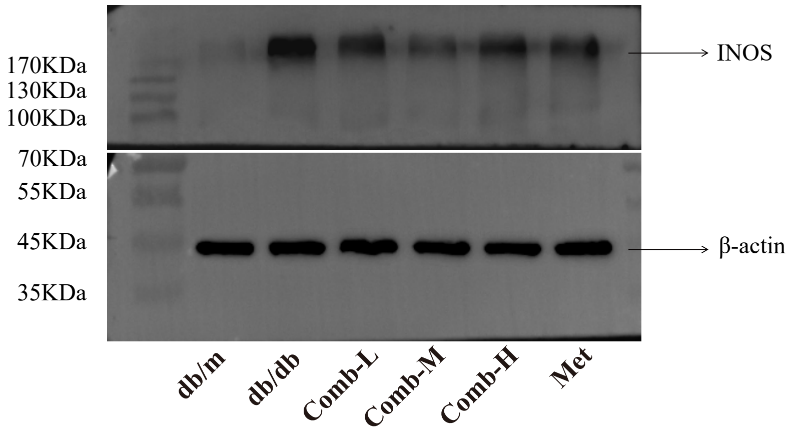

Supplement: Supplementary file 1 [file DataSheet1.pdf]
